# Comparative transcriptome analysis and CRISPR/Cas9 gene editing reveal that E4BP4 mediates lithium upregulation of *Per2* expression

**DOI:** 10.1098/rsob.210140

**Published:** 2021-12-15

**Authors:** Qin Zhou, Kankan Wang, Jiameng Qiu, Di Zhu, Tian Tian, Yunfei Zhang, Ximing Qin

**Affiliations:** ^1^ Department of Health Sciences, Institutes of Physical Science and Information Technology, Anhui University, Hefei, Anhui Province 230601, People's Republic of China; ^2^ Modern Experiment Technology Center, Institutes of Physical Science and Information Technology, Anhui University, Hefei, Anhui Province 230601, People's Republic of China

**Keywords:** bipolar disorder, circadian clock, crispr/cas9, *Per2* expression, lithium

## Abstract

Bipolar disorder (BPD) is a psychiatric disorder characterized by alternate episodes of mania and depression. Disruption of normal circadian clock and abnormal sleep cycles are common symptoms of BPD patients. Lithium salt is currently an effective clinical therapeutic drug for BPD. Animal and cellular studies have found that lithium salt can upregulate the expression of the clock gene *Per2*, but the mechanism is unknown. We aim to understand the mechanism underlying the *Per2* upregulation by lithium treatment. By taking approaches of both comparative transcriptome analysis and comparative qPCR analysis between human and murine cells, Lumicycle assay, luciferase assay and RT-qPCR assay showed that lithium could significantly upregulate the expression of *Per2* in both mouse and human cells, and significantly inhibit the expression of *E4bp4*, which encodes a transcriptional inhibitor of *Per2*. After knocking out the cis-element upstream on the *Per2* promoter that responds to E4BP4, the upregulation effect on *Per2* by lithium disappeared. When *E4bp4* gene was knocked out, the upregulation effect on *Per2* by lithium salt disappeared. This study has found that lithium upregulates *Per2* expression by reducing the expression of transcription factor E4BP4, but the mechanism of lithium salt downregulation of E4BP4 remains to be further studied. Our study provides a new therapeutic target and approaches for treating BPD.

## Graphical abstract

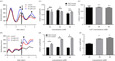

## 1. Introduction

Bipolar disorder (BPD), characterized by episodes of alternate mania and depression phases, is estimated affecting approximately 3% of the world population [1]. BPD patients usually have an early age onset of the first manic/hypomanic or depressive episode, starting at 18.2 years old [1]. During the mania phase, patients have reduced need for sleep, while patients usually suffer from hypersomnia during the depression phase. Multiple factors, including genetic, environmental and psychological factors, contribute to the pathology of this disorder. Among these factors, strong correlations are observed between abnormal circadian rhythms and BPD [2]. Molecular studies have demonstrated there is a phase advance of expression of some main clock genes during the mania phase [3].

The components that constitute circadian rhythms in mammals are conserved, which include core clock proteins and accessorial clock proteins. The core components are composed of positive elements, BMAL1 (Brain and Muscle Arnt-like protein-1) and CLOCK (Circadian Locomotor Output Cycles Kaput), and negative elements, PERIORDs (PER1, PER2 and PER3) and CRYPTOCHROMEs (CRY1 and CRY2) [4,5]. BMAL1 and CLOCK form heterodimer complexes (B/C complexes in the following text) and then activate transcription of *Per* and *Cry* genes. After transcription, translation and post-translational modifications, PERs and CRYs enter the nuclei to repress the transcriptional activity of B/C complexes [6,7]. As PERs and CRYs degrade, the repression function fades away and B/C complexes activate transcription again to start a new circadian cycle. The cycle is approximately 24 h. Accessorial feedback loops include *Rev-erb* [8] and *Ror* [9] and their coding products. REV-ERB and ROR regulate the transcription of the *Bmal1* gene that encodes the BMAL1 protein by associating with RORE elements in opposite directions. E4BP4 and DBP bind to D-box elements to regulate the transcriptional activity of relative genes [5,10].

Human genetic mutations or polymorphisms in these clock components have been suggested to link to BPD, including *BMAL1*, *CLOCK*, *PER2*, *PER3*, *CRY2*, *RORα* and *RORβ* genes [11–17]. Mice that carry a mutation on the *Clock* gene exhibit human mania-like symptoms which can be attenuated by chronic lithium administration [18]. As the most applied medicine to treat BPD, lithium was reported to affect the phosphorylation of GSK-3*β*, which in turn could reduce its kinase activity, resulting in a longer circadian period [19,21]. Yin *et al*. showed that the inhibition of GSK-3*β* by lithium promotes the degradation of the nuclear receptor Rev-erb*α*, leading to increased *Bmal1* expression [22]. However, both GSK-3*β* specific inhibitors and siRNA knock-down of GSK-3*β* caused a short circadian period, in contrast to the effect of lithium [23]. The accessorial clock gene *Dbp* was revealed to be downregulated with chronic lithium treatment of lymphoblastoid cell lines [24]. *Egr1* has been reported to be a molecular target of lithium to enhance *Per2* expression [25]. Iitaka and his colleagues originally reported that the peak level of *mPer2* mRNA increased in the presence of lithium in a dose-dependent manner [19]. A microarray study has shown that *Per2* expression was upregulated in mouse brain with the treatment of lithium carbonate [26]. Recent studies have further demonstrated that lithium induced the expression of *Per2* gene [27,28].

Lithium has been widely prescribed for treatments of BPD patients in current guidelines [29], while most mechanistic studies on lithium related to circadian rhythms were carried out in rodent models [19,25,27]. The mechanisms behind *Per2* upregulation in the presence of lithium remain unknown. In our study, we found that the presence of cycloheximide (CHX), a translation inhibitor, abolished the upregulation of *Per2* by lithium treatment. Therefore, in order to understand the direct target of lithium, we carried out a comparative transcriptome analysis using lithium-treated human cell lines in the absence or presence of CHX. To our knowledge, no comparative studies between human and rodents have been performed to investigate the common circadian gene target of lithium. We hypothesized that lithium may have common targets in cells derived from both human and mouse sources. In this report, we took advantage of comparative approaches, circadian luminescence analysis, luciferase assays and qRT-PCR assays to investigate common targets of lithium in both human and mouse cell lines. Furthermore, we employed CRISPR genomic editing tools to knock-out the *E4bp4* gene, which was coordinately regulated together with *Per2* in our comparative analysis. Collectively, we conclude that lithium upregulates *Per2* expression by reducing the expression of the repressive transcription factor E4BP4.

## 2. Material and methods

### 2.1. Reagents, plasmids and cell lines

U2OS (human osteosarcoma) cell lines stably transfected with either a *BMAL1::dluc* (U2OS-C26) or a *PER2::dluc* (U2OS-D15) reporter [30] were grown in DMEM (Gibco, #11965–092) supplemented with 10% fetal bovine serum and 1% penicillin/streptomycin at 37°C (5% CO_2_). NIH3T3 and U373 cell lines were grown in the same medium. Unless mentioned, the reagents used in this study were purchased from Sangon Biotech, Shanghai, China.

The mouse *E4bp4* (NCBI: NM_017373.3, also known as *Nfil3*) cDNA containing the entire coding region was PCR amplified using NIH3T3 cDNA library with the following primer sets: E4bp4-sense, 5′-CGCGGATCCATGGATTACAAGGATGACGACGATAAGATGCAGCTGAGAAAAATGCAG-3′ (underlined is the *BamH*I site, followed with nucleotides that encode a FLAG tag), and E4bp4-antisense, 5′-CCGCTCGAGTTACCTGGAGTCCGAAGCCG-3′ (underlined is the *Xho*I site). The PCR-amplified fragment was cloned into the pcDNA3.1 vector to generate the expression construct pcDNA-flag-mE4BP4.

pGL3-P*hPER2*-Luc and pGL3-P*mPer2*-Luc reporter constructs were made as follows. m*Per2* promoter a 1109 bp fragment of the mouse *Per2* promoter region (−798 to +311 bp from the transcription start site) was cloned into the pGL3 basic vector (#E1751, Promega, USA), named pGL3-P*mPer2*-Luc, using a forward primer 5′-CCGCTCGAGGATTGAGGGCAGGAAGAAATC-3′ (underlined is the *Xho*I site) and a reverse primer 5′-CCCAAGCTT AACTAATCTCTCCGACCTCAG-3′ (underlined is the *Hind*III site). h*PER2* promoter, a 696 bp fragment of the human *PER2* promoter region (–500 to +196 bp from the transcription start site), was cloned into pGL3 and named pGL3-P*hPER2*-Luc, using a forward primer 5′ CCGCTCGAGTGGAGGTCTCCCTCGTCCGG-3′ and a reverse primer 5′-CCCAAGCTT CAAGCCGAGGAGTCCAGCAG-3′. All the procedures involving the PCR step were confirmed by sequencing to be free of mutations.

### 2.2. Sequence alignment

Promoter sequences of the human *PER2* and mouse *Per2* genes were searched in the Eukaryotic Promoter Database (https://epd.epfl.ch//index.php). In order to find the homology of *Per2* promoter in human and mouse, DNAman software (Lynnon, Quebec, Canada) was used for the sequence alignment.

### 2.3. NIH3T3/*mPer2::luc* cell line

A hygromycin resistance gene cassette (hygroR) was inserted into pGL3-P*mPer2*-Luc, resulting in the reporter construct pGL3-Hygro-P*mPer2*-luc. Cultured NIH3T3 cells were transfected with the construct pGL3-Hygro-P*mPer2*-luc, followed by hygromycin screening at 300 µg ml^−1^. After a population of hygromycin-resistant cells was successfully obtained, monoclonal cell lines were screened by the limiting dilution method. Luminescence recording assays were performed with monoclonal cell lines to confirm that *mPer2* reporter was stably transfected into NIH3T3 cells. The 2F5 monoclonal cell line was used in this study.

### 2.4. Circadian luminescence recording assay

1.5 × 10^5^ U2OS *PER2*-Luc (U2OS-D15) or U2OS *BMAL1*-Luc (U2OS-C26) cells were inoculated into 35 mm culture plates (Costar, #9102) in triplicate. On the next day, cells in each well were synchronized with 2 h treatment of 100 nM dexamethasone and recorded in a Lumicycle as described previously [31]. Different concentrations of lithium chloride (LiCl) were added at peaks of the luminescence oscillation, using sodium chloride (NaCl) as the vehicle control. Bioluminescence data were analysed with the Lumicycle analysis program (Actimetrics, USA) to obtain circadian parameters such as period and amplitude.

### 2.5. Dual luciferase assay to assess the activity of lithium chloride

NIH3T3 cells were seeded into six-well plates, and 1 µg promoter reporter constructs of pGL3-PhPER2-Luc and pGL3-PmPer2-Luc were separately transfected into the cells using Lipo6000 (Beyotime, Beijing, China), using a Rluc reporter as reference; 24 h post-transfection, 20 mM LiCl or NaCl was added into the media for 8 h, then the levels of firefly luciferase activity were measured sequentially using Dual Luciferase Reporter Assay Kit (Beyotime, China).

### 2.6. Cycloheximide treatments to inhibit protein translation

U2OS-D15 cells were plated in the 60 mm dishes, and then 10 µM CHX (solved in DMSO) was added into each dish at the peaks of the luminescence oscillation, using NaCl as the vehicle control. One hour later, 20 mM LiCl was added in the CHX-treated and DMSO-treated groups, each group using the 20 mM LiCl as the control.

### 2.7. RNA-seq analysis

U2OS-D15 cells were simultaneously inoculated into 60 mm and 35 mm Petri dishes for culture. When the number of cells reached 60% confluency, the cells were synchronized with dexamethasone for 2 h. The cells in the 60 mm dish continued the culture, while the cells in the 35 mm dish were recorded by the Lumicycle in the presence of fluorescein. When the Per2-Luc oscillation in the cells was about to reach its peak, CHX was added to the 60 mm Petri dish for 1 h, then 20 mM LiCl (NaCl as the control) was added for 4 h, and the cells were collected into Trizol reagent. The collected cells were sent to Personal Biotechnology in Shanghai for RNA-seq. Three biological replicates were carried out for this experiment. The cDNA library of each sample was prepared and sequenced by Personal Biotechnology. Using the second-generation sequencing technology, based on the Illumina sequencing platform, double-terminal sequencing of these libraries was carried out. FastQC software is used to analyse the quality control of the preprocessed data. Based on the human reference, genome sequence downloaded from the Ensembl database (Homo_sapiens. GRCh38). The filtered Reads were compared to the reference genome using HISAT2 software. We used HTSeq to compare the Readcount value of each gene, and FPKM was used to standardize the expression. We used DESeq to analyse the difference of gene expression. Those genes with a false discovery rate < 0.1 were considered as differentially expressed between control and exposed groups, and genes with |log^2^ (Fold_change)|> ± 0.5 were considered with a relevant biological signal. We use topGO for GO enrichment analysis. In the analysis, we used the differential genes annotated by GO term to calculate the gene list and the number of genes in each term and find out the GO term, in which the differential genes are significantly enriched compared with the whole genome background, so as to determine the main biological functions of the differential genes.

### 2.8. Site-directed mutagenesis

The B-site and E2-site were, respectively, mutated by amplifying the whole plasmid of the pGL3-P*hPER2*-Luc using a high-fidelity Taq polymerase. A pair of complementary primers 5′-GGCCCCGCTGCGGCCAGATCTCCCGCCGCCGGC-3′ and 5′-GCCGGCGGCGGGAGATCTGGCCGCAGCGGGGCC-3′ were used to mutate the B-site 5′-CTTACGTAACC-3′. Primers 5′-CGGTCACGTTTTCCACGCTAGGAACAGCGGCGA-3′ and 5′-TCGCCGCTGTTCCTAGCGTGGAAAACGTGACCG-3′ were used to mutate the E2-site 5′-TCCACTATGTGAACAG-3′. After the amplification, we used *Dpn*I (New England Biolabs) to digest the template plasmids. Then, the product was transformed into the DH5*α* competent cells. To obtain the site-directed mutagenesis plasmids, the plasmids from the transformed *E. coli* colonies were sent to sequencing for identification.

### 2.9. Quantitative real-time PCR to assess gene expression

U2OS-D15 cells and NIH3T3-Per2 cells in 60 mm dishes were synchronized for 2 h, and then the cells were treated with 20 mM LiCl (NaCl as the control) for 4 h since the peak of circadian oscillation. Total RNA was extracted from cells using the RNeasy kit (Qiagen, #74104), according to the manufacturer's instructions. One microgram of total RNA was reverse-transcribed to cDNA using HiScript III RT SuperMix for qPCR (+gDNA wiper) (Vazyme, #R323–01, Nanjing, China) according to the manufacturer's instructions. SYBR green fluorescence-based qRT-PCR was performed for RNA quantification (#Q511-02, Vazyme, China). The Delta-Delta-Ct method was used for the normalization of qPCR results. *β-actin* and *rplo* were used as the internal control to normalize the gene expression level of NIH3T3 and U2OS cells, respectively. The primer sets used in this study to quantify both human and mouse clock gene expressions were listed in the electronic supplementary material, tables S1 and S2.

### 2.10. Immunofluorescence microscopy

NIH3T3 cells were grown as described in above section. A sterile cover glass was put in each well and 3 × 10^5^ cells were seeded per well 1 day before transfection. When cells grew up to 70–80% confluency, they were transfected with pcDNA-flag-mE4BP4 using Lipo6000 (Beyotime, China). Twenty-four hour after transfection, cells were fixed in 4% paraformaldehyde. Then, cells were permeabilized and blocked with 0.3% TritonX-100, 2% BSA, 0.05% Tween in PBS for 1 h at room temperature, followed by sequential washing with PBS and were subjected for incubation using FLAG antibody. The second antibody was a Cy3-conjugated rabbit anti-mouse IgG (#D110172, BBI, Shanghai, China). Cells were embedded in Mounting Medium, Antifading (S2110, Solarbio). Immuno-labelled cells were analysed using immunofluorescence microscopy (TCS SP8, Leica).

### 2.11. Immuno-blotting assay

Cells were harvested in lysis buffer and mixed with reduced loading sample buffer, followed by separation by 10% SDS-PAGE (60 V for 30 min, 120 V for 60–90 min). The gel was then transferred onto nitrocellulose filter (NC) membranes and blocked in 10% skim milk in PBS-Tween (PBST) for 1 h at room temperature. Antibodies for Per2 (PER21-A; Alpha Diagnostic International) and tubulin (KM9007; Tianjin Sungene Biotech) were used. Cy3-conjugated rabbit anti-mouse antibodies (#D110172, BBI) were used as the secondary antibodies. Immunoreactive bands were detected by Typhoon laser scanners (FLA-9500, GE, USA).

### 2.12. CRISPR/Cas9 technology to knock-out m*E4bp4* gene in NIH3T3 cells

Oligonucleotides specific for the second exon of m*E4bp4* genomic locus was designed using the Optimized CRISPR Design tool (http://crispr.mit.edu/) [32]. The synthesized oligonucleotides (detailed sequence present in the text) were annealed, dephosphorylated and then ligated into the expression vector pX459 (pSpCas9(BB)-2A-Puro was a gift from Feng Zhang, Addgene plasmid # 48139) using *Bbs*I restriction site. NIH3T3 cells were seeded into six-well plates 1 day before transfection at a density of 2 × 10^5^ cells per well. Cells were transfected with the construct targeting *E4bp4* gene locus using Lipo6000 reagents (#C0529, Beyotime, China) according to the manufacturer's instruction. Twenty-four hours after transfection, cells were screened with 1 µg ml^−1^ puromycin. Monoclonal cell lines were screened by the limiting dilution method. The genomic information of the monoclonal cell lines were further confirmed by DNA sequencing.

### 2.13. Statistical analysis

The data are presented as the mean ± standard deviation (s.d.). All experiments were repeated at least three times. The Statistical Package for the Social Sciences (IBM Corporation, Armonk, NY, USA) was used to assess significant differences. Data were analysed using a two-tailed Student's *t*-test. *p <* 0.05 was considered statistically significant.

## 3. Results

### 3.1. Lithium treatment upregulates the luminescence that is under the control of *PER2* promoter

**Figure 1.**
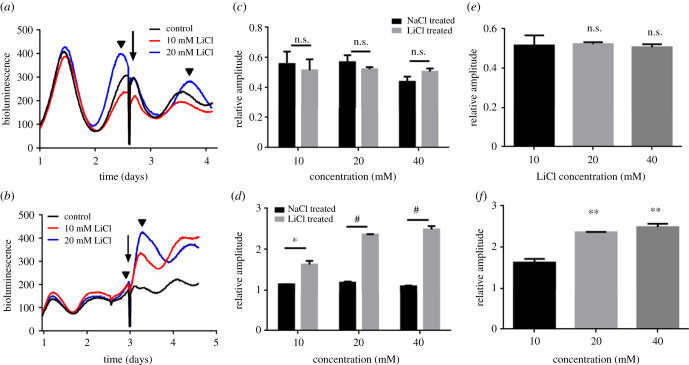
Lithium treatment of U2OS cells upregulates luminescent reporters that are under the control of *PER2* promoter. (*a*) U2OS-C26 cells harbouring the firefly luciferase reporter under the control of *BMAL1* promoter were treated with different concentrations of LiCl at the peak of luminescence. Representative traces are illustrated. (*b*) U2OS-D15 cells harbouring the firefly luciferase reporter under the control of *PER2* promoter were treated with lithium. (*c*) Relative amplitudes of luminescence under the control of *BMAL1* promoter were quantified at different concentrations of lithium, along with corresponding concentrations of sodium as the control. Relative amplitude was defined as comparing the peak levels (labelled with black triangles in (*a*) and (*b*)) of luminescence after and before the treatments. (*d*) Relative amplitudes of luminescence under the control of *PER2* promoter were quantified. (*e*) There was no significant difference of luminescence in C26 cells among different concentrations of lithium. (*f*) Clear dose-dependent upregulation of luminescence in D15 cells when lithium concentration increases. Experiments were carried out at least six independent times. Error bars represent the mean ± s.d. (n.s., no significance; ^#^*p* < 0.0001; ***p < 0.001; **p < 0.01).

Human osteosarcoma U2OS cells were engineered to express firefly luciferase reporters under control of *BMAL1* promoter (U2OS-C26) and *PER2* promoter (U2OS-D15), respectively, as described above. In order to exhibit the effect of lithium on the expression of luminescent reporters, LiCl was added during the second or third cycle instead of at the beginning of the experiments, using NaCl as the control. Furthermore, since the rising phase of the luminescence may interfere with the upregulation effect, lithium was treated at the peak of luminescence, as determined by monitoring the luminescent reporters (figure 1*a,b*). As presented in figure 1, lithium treatments acutely enhanced the luminescence in the D15 cells, but not in the C26 cells. Like control traces, adding lithium did not affect the trend of luminescence in C26 cells (figure 1*a*). However, for D15 cells, adding lithium suddenly raised the luminescence trace while the control trace was still in its falling trend (figure 1*b*). Different concentrations of lithium (10, 20 and 40 mM) were used for the experiments along with corresponding sodium controls (figure 1*c,d*). The effect of lithium was measured by relative amplitudes that were defined as comparing the peak levels (labelled with black triangles) of luminescence after and before the treatments (figure 1*a,b*). We observed that the relative amplitude of the luminescence under the control of *Per2* promoter was upregulated in a dose-dependent manner (figure 1*f*), but not for these C26 cells (figure 1*e*). Also, the overall baseline of the luminescence was high under the treatments of lithium in the D15 cells (figure 1*b*). We further tested the consequence of treating D15 cells with lithium at the trough of luminescence, and we observed higher-luminescence peaks after the treatments, which is also dependent on the lithium concentration (electronic supplementary material, figure S1). These data indicate that lithium specifically enhances the activity of *Per2* promoter, but not that of *Bmal1* promoter. The basal level of luminescence under the control of *Per2* promoter is not affected when the D15 cells were either treated with NaCl or PBS (electronic supplementary material, figure S2). Thus, we chose NaCl as a control in the following experiments.

### 3.2. Inhibition of protein translation blocked the upregulation of *PER2* by lithium

**Figure 2.**
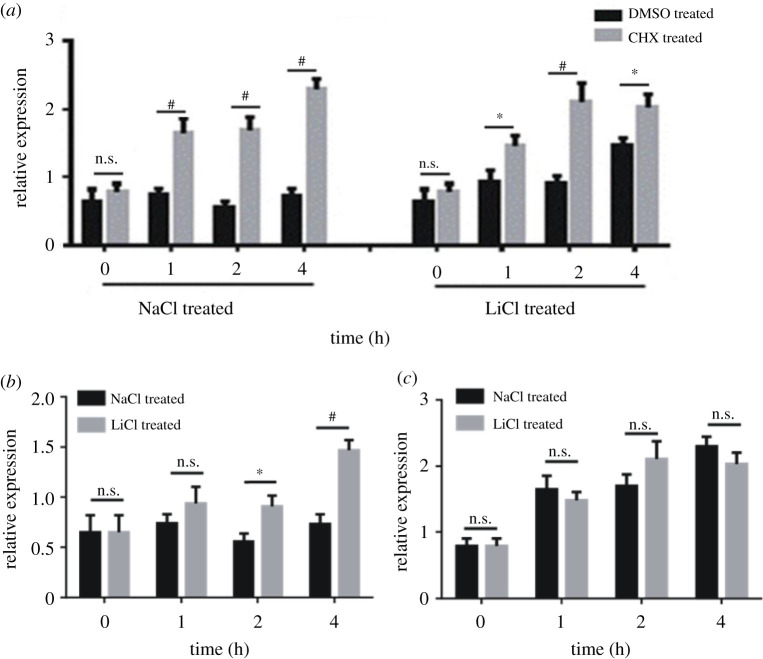
Translation inhibitor CHX abolished *PER2* upregulation by lithium. (*a*) DMSO or CHX was incubated with U2OS-D15 cells 1 h before adding sodium or lithium. The expression level of *PER2* was quantified at time 0, 1, 2 and 4 h after adding sodium or lithium. *PER2* levels were accumulating in both sodium and lithium groups. (*b*) In the DMSO control, *PER2* level was upregulated when cells were treated with lithium. (*c*) In the presence of CHX, protein translation being inhibited, *PER2* level showed no difference between sodium and lithium groups at any time point. Error bars represent the mean ± s.d. (n.s., no significance; ^#^*p* < 0.0001; *p < 0.05).

Multiple signalling pathways are capable of activating circadian clock gene expressions both *in vitro* and *in vivo* [33,34]. We wished to determine whether signalling pathways are involved in activating *PER2* expression under lithium treatments. If any signalling pathway that can directly target a specific response element on the promoter of *PER2* was activated by lithium, inhibition of translation would not prevent the upregulation of *PER2* expression. DMSO or 10 µM protein synthesis inhibitor CHX was added to the human U2OS-D15 cells 1 h before the lithium treatment. Then, 20 mM NaCl or 20 mM LiCl was employed. In spite of being treated with sodium or lithium, *PER2* expressions were accumulating over time in the presence of CHX, more than the DMSO controls (figure 2*a*). *PER2* transcripts showed a significant difference within 1 h when protein synthesis was blocked by CHX, leading to accumulated RNA. Some other clock or clock-regulating genes were also quantified under this condition, such as *DEC1*, *DEC2* and *EGR1* genes. Transcripts of these genes accumulated as well in the presence of CHX (electronic supplementary material, figure S3).

When cells were incubated with DMSO, 20 mM LiCl apparently upregulated *PER2* expression at time points 2 h and 4 h (figure 2*b*). In this experiment, time 0 indicated the peak of the *PER2* expression, since the luminescence was monitored under the lumicycle detector. When the translation inhibitor CHX was applied, transcripts of *PER2* were quickly accumulating for both sodium and lithium groups, and there was no difference of *PER2* level between two groups at any time point (figure 2*c*). Thus, lithium treatment cannot directly activate signalling pathways that could target cis-regulating elements on the *PER2* promoter region to upregulate its expression. Transcription factors have to be involved in the phenomenon of *PER2* upregulation by lithium. Our observations indicated that there is a factor which was directly affected by lithium transcriptionally and its translation product can regulate *PER2* expression. Therefore, we conducted comparative transcriptome analysis of the U2OS-D15 cells in the absence or presence of CHX under the treatment of LiCl.

### 3.3. Comparative transcriptome analysis indicates that E4BP4 may be the direct target of lithium

**Figure 3.**
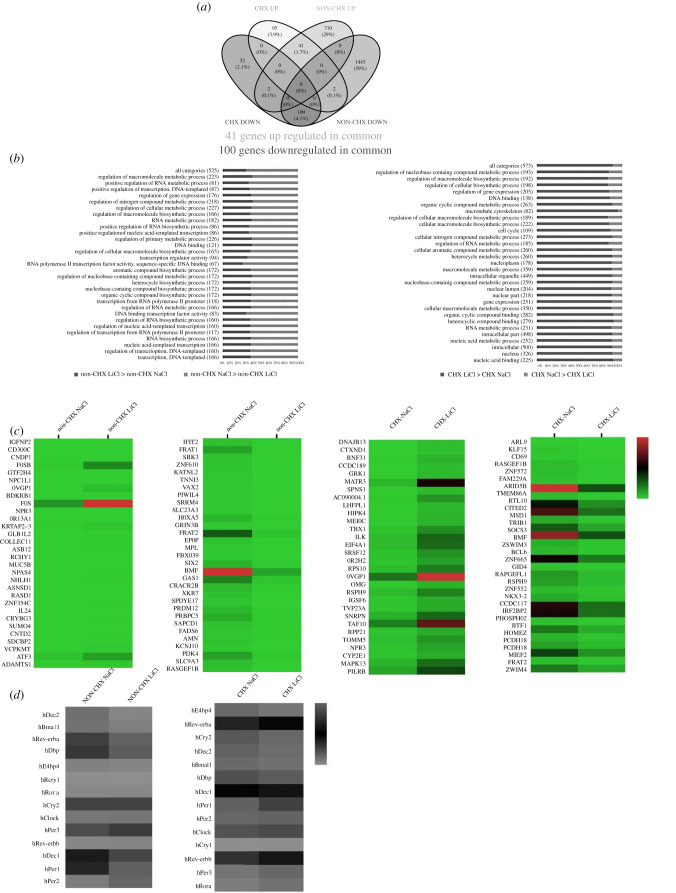
Comparative transcriptome analysis of U2OS cells treated with lithium in the presence or absence of CHX. (*a*) Venn diagram showing the numbers of transcripts in U2OS cell that were significantly increased or decreased in expression by 20 mM lithium in the presence or absence of CHX. (*b*) Differential expression patterns between non-CHX and CHX after being treated with lithium for major functional categories. The bracketed numbers to the right of the category names indicate the range in the number of genes identified for that category. Blue bars signify the percentage of genes with higher expression in LiCl group than NaCl group, while red bars signify the percentage of genes with higher expression in NaCl group than LiCl group (*p* < 0.005). (*c*) Heatmap of top 30 differentially expressed genes comparing transcription levels among samples of non-CHX and CHX groups (NaCl V.S. LiCl). (*d*) The heatmap of biological clock gene expression in non-CHX and CHX groups (NaCl V.S. LiCl).

U2OS-D15 cells were treated with CHX for 1 h before adding LiCl or NaCl, which was described in the Material and methods section. Four hours after LiCl (NaCl as the control) treatment, cells were collected and the transcriptomes under both treatments were analysed by RNA-seq. Based on the Illumina sequencing platform, the transcriptome sequencing of the samples was carried out. The average raw data of each sample was 6G, and the range of original reading times was 6356378700-7367250600. Sequencing readings that contain low quality, joint contamination and high content of unknown base (N) readings were removed. The gene differentiation analysis was carried out by DESeq, and the screening condition was that the gene expression was upregulated by 150% and downregulated by 75%, and the significant *p*-value was less than 0.1. Based on the analysis of differential expression of genes in the non-CHX group (LiCl versus NaCl) and the CHX group (LiCl versus NaCl), 2298 differentially expressed genes (753 upregulated and 1545 downregulated) and 292 differentially expressed genes (138 upregulated and 154 downregulated) were detected, respectively (figure 3*a*).

GO enrichment analysis of differentially expressed genes was carried out by topGO, and the top 30 GO terms with the most significant enrichment were selected for functional analysis (figure 3*b*). We found that the differentially expressed genes are mainly related to metabolic regulation, transcriptional regulation and biosynthesis. In addition, we analysed the top 30 genes of upregulated and downregulated genes in the non-CHX and CHX groups of samples (figure 3*c*). In the non-CHX group, *PER2* was upregulated about 2.5 change folds after LiCl treatment, while in the CHX group, the upregulation of *PER2* disappeared after LiCl treatment. Through the analysis of the common changed 141 genes (41 upregulated, 100 downregulated) between the non-CHX and CHX groups (figure 3*a*), in the common downregulation set, we found that the *E4BP4* was downregulated about 0.65 change folds in both groups. We demonstrated the heatmap of expression values for the 14 most important biological clock genes (figure 3*d*). Among these genes, *PER2* was upregulated only in the non-CHX group, while *E4BP4* was downregulated in both groups. These results indicate that LiCl can upregulate the expression of *PER2* in cells, but when CHX is used to block the translation pathway, the upregulation of *PER2* expression disappears. However, the presence of CHX did not affect the downregulation of *E4BP4* by lithium.

### 3.4. Gene expression patterns of the circadian clock or related genes after the treatment of lithium in U2OS cells

Most mammalian circadian clock genes were discovered during the past few decades, and the clock runs rhythmically under the control of transcriptional and translational feedback loops. *PER* genes, including three homologous members, stand in the main path of this system [5]. Positive transcriptional factors BMAL1 and CLOCK form heterodimers and then activate the gene transcription of *PER* genes through associating with the E-box that locates upstream to their promoter regions, including the *PER2* gene. After transcription and translation, PER proteins form complexes with key clock components CRY1/CRY2, therefore inhibiting the activation of BMAL1/CLOCK complexes. Other regulatory clock components were discovered, including several pairs that play opposite roles during transcription, such as REV-ERBs and RORs, DBP and E4BP4, and the DEC family. Since lithium can enhance the luminescence expression that is under the control of an exogenous *PER2* promoter in U2OS cells (figure 1*b*), we aimed to verify that the endogenous *PER2* was indeed upregulated by the treatment of lithium besides using the RNA-seq technique. Furthermore, these main clock genes and the clock-related genes that play regulatory functions in transcription were examined under the lithium treatment situation in U2OS cells.

**Figure 4.**
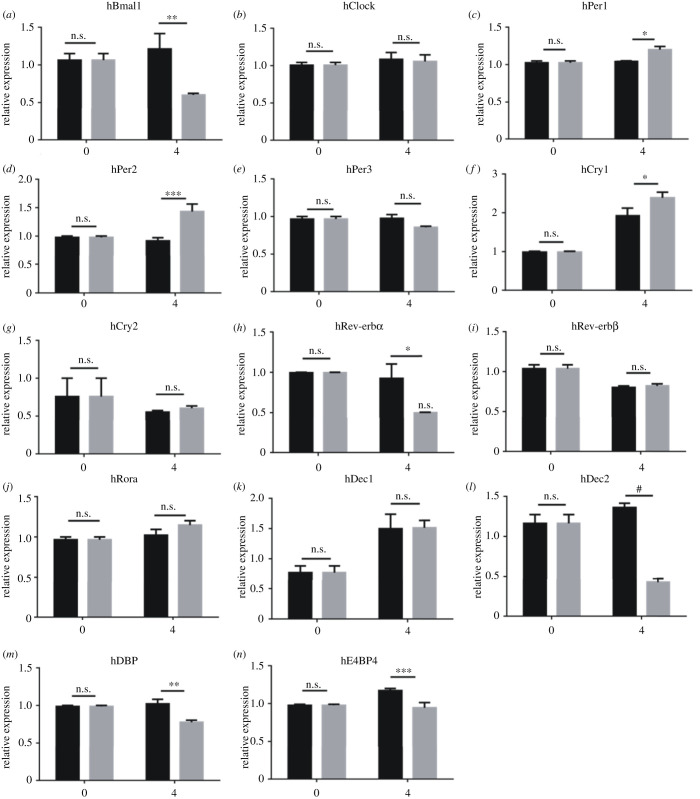
Expression levels of main clock genes and related regulatory genes in U2OS cells treated by lithium. Tested genes are labelled on top of each panel. Black bars indicate the 20 mM sodium controls, and grey bars indicate the 20 mM lithium experiment groups. *BMAL1*, *REV-ERBα*, *DEC2*, *DBP* and *E4BP4* exhibited downregulation, while *PER1*, *PER2* and *CRY1* showed upregulation. Experiments were carried out with triplicates. Error bars represent the mean ± s.d. (n.s., no significance; ^#^*p <* 0.0001; ***p < 0.001; **p < 0.01).

U2OS-D15 cells were treated with 20 mM LiCl at the luminescence expression peak time, which can be determined by monitoring the luminescence. We measured expression levels of *BMAL1*, *CLOCK*, *PER1/2/3*, *CRY1/2*, *REV-ERBα/β*, *RORα*, *DEC1/2*, *DBP* and *E4BP4* genes at 0 and 4 h after the lithium treatment. Compared to the sodium controls, expression levels of *PER1*, *PER2* and *CRY1* were upregulated, while those of *BMAL1*, *REV-ERBα*, *DEC2*, *DBP* and *E4BP4* were downregulated in U2OS cells (figure 4). For *PER2* gene, the expression level increased to 1.5-fold compare to its sodium control (figure 4*d*). The remaining tested genes, *CLOCK*, *PER3*, *CRY2*, *REV-ERBβ*, *RORα* and *DEC1*, were not affected. The upregulation of endogenous *PER2* confirmed that lithium can activate its expression through regulating its promoter region.

### 3.5. Gene expression patterns of the circadian clock or related genes after the treatment of lithium in NIH3T3 cells

We wondered if *Per2* was upregulated in cells from the most commonly used animal model, the house mouse. We chose NIH3T3 cells since these cells have robust circadian oscillations, as demonstrated elsewhere [35]. In order to be consistent with the treatment as done in U2OS cells, we need to know the expression peak of *Per2* gene in NIH3T3 cells. The firefly luciferase reporter NIH3T3 cells were successfully engineered by transfecting them with *mPer2* promoter reporter constructs, as explained above. Our NIH3T3-Per2 monoclonal cell lines showed robust circadian oscillations of firefly luminescence expression, which allow us to add lithium at the peak of luminescence (electronic supplementary material, figure S4). The monoclonal cell line 2F5 was used in this study.

**Figure 5.**
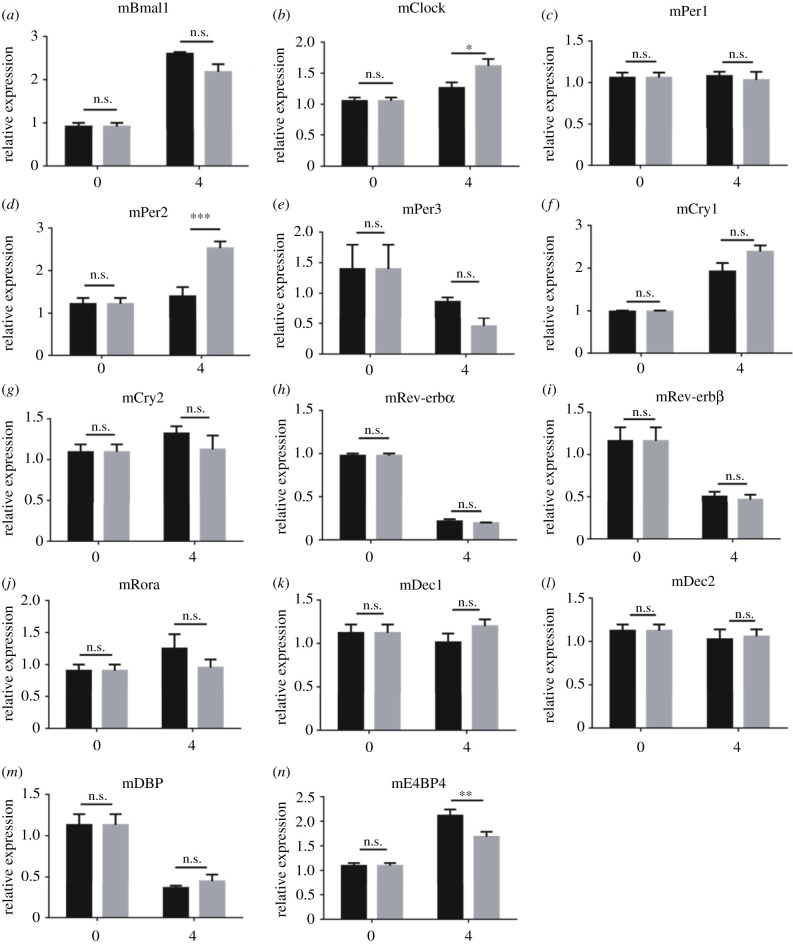
Expression levels of main clock genes and related regulatory genes in NIH3T3 cells treated by lithium. Tested genes are labelled on top of each panel. Black bars indicate the 20 mM sodium controls, and grey bars indicate the 20 mM lithium experiment groups. *Clock* and *Per2* were upregulated, while only *E4bp4* was downregulated. Experiments were carried out with triplicates. Error bars represent the mean ± s.d. (n.s., no significance; **, *p <* 0.01).

Then, we treated NIH3T3-2F5 cells with 20 mM LiCl, using NaCl as controls. The genes that are homologous to those being tested with U2OS cells were measured at the same time points within NIH3T3-2F5 cells. In the case of using mouse-derived NIH3T3 cells lines, different gene expression patterns were observed. Compared to the sodium controls, expression levels of *Clock* and *Per2* were upregulated, while only *E4bp4* was downregulated in NIH3T3 cells (figure 5). For the genes that were differentially regulated in human U2OS cells, such as *Per1*, *Cry1*, *Bmal1*, *Rev-erbα*, *Dec2* and *Dbp*, their expression levels remained unaffected (table 1). For *Per2* gene, the expression level increased to twofold compared to its sodium control (figure 5*d*). This comparative study helps us exclude the possibility that lithium upregulates *Per2* through the mainstream regulating positive factors, BMAL1/CLOCK complexes. Also, the comparative study between human and mouse cells (figures 4 and 5, and table 1) is consistent with the comparative transcriptome analysis (figure 3) that *Per2* was upregulated and *E4bp4* was downregulated by lithium.

**Table 1.** Circadian clock gene expression patterns in human and mouse cell lines.

human			mouse		
	upregulate	downregulate		upregulate	downregulate
*hPer1*	*		*mPer1*		
*hPer2*	***		*mPer2*	***	
*hPer3*			*mPer3*		
*hCry1*	*		*mCry1*		
*hCry2*			*mCry2*		
*hE4BP4*		***	*mE4BP4*		**
*hBmal1*		**	*mBmal1*		
*hClock*			*mClock*	*	
*hDBP*		**	*mDBP*		
*hRev-erbA*		*	*mRev-erbA*		
*hRev-erbB*			*mRev-erbB*		
*hRorα*			*mRorα*		
*hDec1*			*mDec1*		
*hDec2*		***	*mDec2*		

### 3.6. Transient luciferase reporter experiments support that lithium upregulates *Per2* expression through the promoter region

Our comparative transcriptome analysis indicated that *E4bp4* might be the direct target of lithium and *Per2* was not directly activated (figure 3). On the other hand, comparative analysis on the clock and related regulatory genes between human U2OS cells and mouse NIH3T3 cells has revealed that only expression changes of *Per2* and *E4bp4* are in common (figures 4 and 5). Meanwhile, we tested whether lithium can upregulate *Per2* and downregulate *E4bp4* in a neuronal cell line, U373 cells. Consistently, we found that *Per2* was upregulated with lithium treatments (electronic supplementary material, figure S5*a*), while *E4bp4* was downregulated with lithium treatments in U373 cells (electronic supplementary material, figure S5*b*). Next, we tested *E4BP4* expression under the treatments of LiCl or NaCl in the presence of CHX in U2OS cells. A significant decrease of *E4BP4* expression was observed at time point 2 h (electronic supplementary material, figure S6*b*). When the translation inhibitor CHX was applied, E4bp4 was also accumulating in the first 2 h in the presence of CHX (electronic supplementary material, figure S6*a*), but its level was significantly lower at 4 h under the lithium treatment (electronic supplementary material, figure S6*c*). In our observation, even without *de novo* synthesis of new transcription factors, *E4bp4* was downregulated to a similar level as in the absence of CHX (figure 4; electronic supplementary material, figure S6). For other genes being tested, *Dec1*, *Dec2* and *Egr1* also decreased to some extent when treated with lithium in the presence of CHX (electronic supplementary material, figure S7).

**Figure 6.**
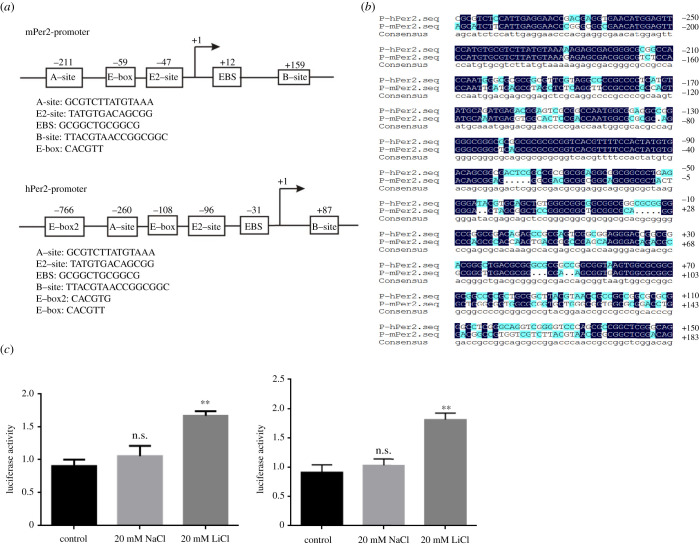
Lithium can activate the *Per2* promoter in a transient transfection experiment. (*a*) Various cis-elements on both mouse *Per2* and human *PER2* promoter regions are labelled and presented with their sequences. The positions of these cis-elements were also labelled, as the transcription site was labelled as +1. (*b*) Alignment between the mouse *Per2* and the human *PER2* promoter regions. Numbers listed on the right are relative to the transcription start site. (*c*) 20 mM LiCl activates *Per2* promoters from both origins. Experiments were carried out with triplicates. Error bars represent the mean ± s.d. (n.s., no significance; **, *p <* 0.01).

As a transcription factor, E4BP4 is known to repress *Per2* expression when it binds to separate DNA elements upstream the *Per2* promoter region. Two E4BP4-binding sites were reported locating on the *Per2* promoter region, B-site and E2-site, respectively [36]. According to the promoter sequences in Eukaryotic Promoter Database (https://epd.epfl.ch//index.php), we constructed a firefly luciferase reporter under the control of *mPer2* promoter region from −798 to +331 sites relative to the transcription start site, and another reporter under the control of *hPer2* promoter region from −500 to +196 sites. These cis-elements on both the mouse *Per2* and the human *PER2* promoter regions were identified and labelled, as presented in figure 6*a*. The DNA sequences from both promoter regions are highly conserved, especially in these cis-element regions (figure 6*b*). We transiently transfected NIH3T3 cells with both reporter constructs, and then 20 mM LiCl or NaCl was added 24 h after transfection. Both firefly luminescence reporters driven by *Per2* promoters were strongly induced when the cells were treated with 20 mM lithium (figure 6*c*). The induction levels of both reporters are equivalent to each other, less than twofold (figure 6*c*). This effect by lithium on transiently expressed reporters is also consistent with the effects observed both from stably expressed reporter cell lines and from endogenous *Per2* expression experiments.

### 3.7. The effect of lithium on the activation of *Per2* expression was mediated by E2-site which is an E4BP4-binding cis-element

**Figure 7.**
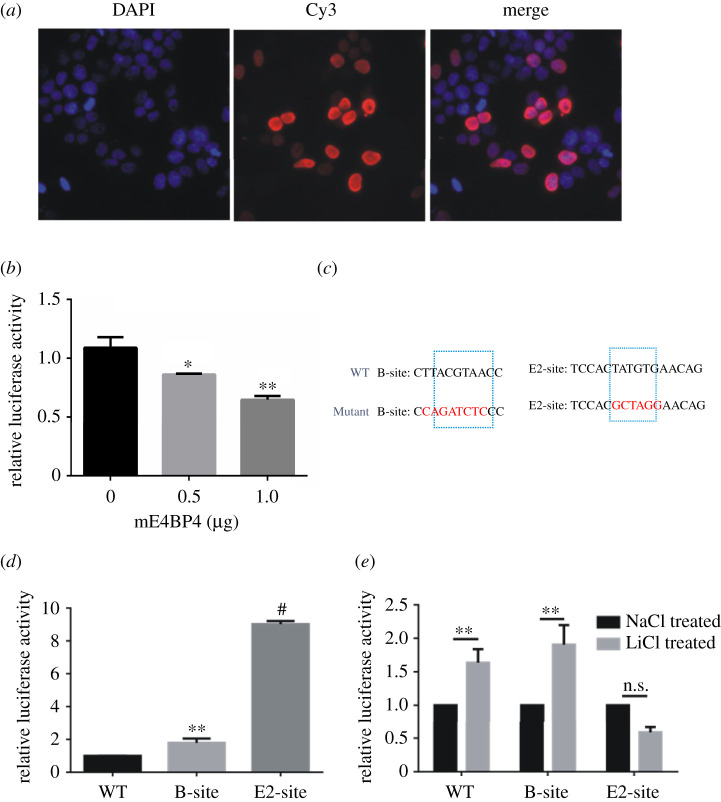
Mutation of E2-site on *Per2* promoter withdrew the effect of lithium on activating *Per2* expression. (*a*) Immunofluorescence staining images indicate exogenous expressed mE4BP4 localized to the nuclei. Nuclei were stained with DAPI (blue), and FLAG-tagged E4BP4 was stained with Cy3-labelled antibody (red). Merge images show that mE4BP4 localized in the nuclei. (*b*) 0, 0.5 and 1 µg of mE4BP4 constructs were co-transfected with the mouse *Per2* luciferase reporter into NIH3T3 cells, respectively. The luciferase expression was repressed in a dose-dependent manner as E4BP4 increased. (*c*) WT and mutant sequences of B-site and E2-site are presented. (*d*) Both B-site mutant and E2-site mutant can enhance the luciferase activity compared to the WT reporter construct. E2-site mutation enhanced the activity up to eightfold higher. (*e*) B-site mutant did not affect the lithium effect on activating *Per2* promoter, while E2-site mutant abolished the effect. Experiments were carried out with triplicates. Error bars represent the mean ± s.d. (n.s., no significance; **p < 0.01).

The amplitude of *Per2* expression can be suppressed by E4BP4 [36,37]. We hypothesized that the upregulation effect of lithium is mediated by downregulated E4BP4 based on our comparative analysis between human and mouse cells. N-terminal FLAG-tagged E4BP4 was expressed in NIH3T3 cells and immunofluorescence staining experiments confirmed that E4BP4 localizes to the nuclei (figure 7*a*), where a transcription factor can function. Then we over-expressed E4BP4 together with transiently expressed mouse *Per2* luciferase reporter in NIH3T3 cells. As the amount of E4BP4 plasmid constructs increased, the luciferase activity was repressed in a dose-dependent manner (figure 7*b*). Among these cis-elements on the *Per2* promoter region, B-site and E2-site are where E4BP4 functions as a transcriptional repressor. We mutated these two sites separately on the mouse *Per2* luciferase reporter construct into the sequences as shown in figure 7*c*. From the results of transient transfection experiments, the mutation of E2-site greatly enhanced luciferase activity up to eightfold higher, which is most likely to be due to a higher luciferase expression level (figure 7*d*). In comparison, the mutation of B-site slightly enhanced the luciferase activity by 1.9-fold (figure 7*d*). We further examined the lithium effect on these two mutant reporters. Like our WT control that lithium can enhance the luciferase activity, lithium-treated cells showed 1.8-fold higher luciferase activity with the B-site mutant reporter; by contrast, the upregulation effect by lithium was withdrawn when the E2-site mutant reporter was used in the assay (figure 7*e*). These results supported our hypothesis that E4BP4 mediates lithium upregulation of *Per2* expression.

### 3.8. Lithium cannot upregulate *Per2* expression in a cell line in which *E4bp4* gene was knocked out using CRISPR/Cas9 genomic editing tools

**Figure 8.**
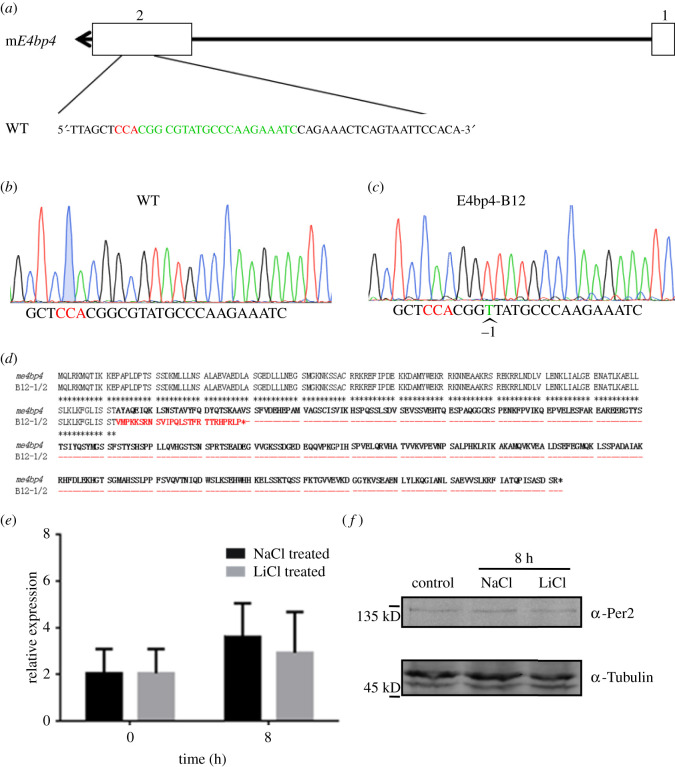
E4bp4 mediates the effect of lithium that upregulates *Per2* expression. (*a*) Schematic of the mouse *E4bp4* locus that is composed of two exons. The gRNA target region was designed to a TGG site in exon 2, whose detailed sequences were presented. (*b*) Sanger sequencing data of the wild-type sequence of the target site. The TGG site is highlighted in red. (*c*) The gRNA target region mutated from CG to T at the -4 position to the TGG site, which caused a frame shift mutation. (*d*) The mutation caused a codon shift in the *E4bp4* locus, resulting in an early stop codon in the translating product. (*e*) LiCl did not induce *Per2* expression in E4bp4 knock-out cell strain. Experiments were carried out with triplicates. (*f*) Per2 protein level was unaffected in the *E4bp4* KO cells when treated with lithium, compared to the sodium control. Tubulin was used as a loading control for the western blotting data.

Mouse *E4bp4* gene has two exons on the chromosome and a 20 nt guide sequence targeting exon 2 was designed according to the CRISPR Design Tool [32], as presented in figure 8. The Cas9 nuclease normally produces a double-strand break at the −3 position relative to the 5′ TGG PAM site. Mouse NIH3T3 cells were transfected with the CRISPR construct that targets exon 2 on the *E4bp4* locus. Stably transfected cells were screened afterwards and one single-colony cell was named E4bp4-B12 cell strain. Using genomic DNA extracted from E4bp4-B12, flanking regions around the target PAM site were PCR amplified and cloned into bacterial vectors and followed with DNA sequencing. Sequencing results from over 15 clones gave us only one result that −4 position to the PAM site was changed from CG to T (figure 8*b,c*). The consistent results indicate that both endogenous alleles were simultaneously mutated into the same sequence. This mutation caused codon shift in the open reading frame of E4BP4, resulting in a STOP codon shortly after the modification site (figure 8*d*). Since the target site is at one-third of the coding sequence, we concluded that even a truncated protein was made, it would not function normally. Next, we tested the effect of lithium on this single-colony cell E4bp4-B12. Twenty millimolar LiCl was treated to cultured cells and the expression levels of *Per2* were quantified by qRT-PCR. The results showed that *Per2* was not induced in this background (figure 8*e*), while *Per2* was greatly upregulated in the wild-type cells. We further examined the Per2 protein level within this cell strain when the cells were treated with lithium, compared to its sodium control. No significant difference could be detected by the western blotting results (figure 8*f*).

## 4. Discussion

By taking comparative transcriptome analysis between CHX and non-CHX conditions, we found that among the 141 common direct regulated genes under the treatment of LiCl, E4bp4 might be the responding transcription factor that was involved in the *Per2* upregulation by lithium. Next, we show from a comparative study that lithium treatment leads to induced expression of *Per2* in both human and mouse cells. Fifteen circadian clock and clock-related genes were examined in both cell lines and *E4bp4* gene expression was also influenced by lithium treatment. E4BP4 functions as a transcription factor that represses many gene expressions, including the *Per2* gene [36,37]. Conserved cis-regulatory elements were identified by aligning the promoter region of both human and mouse *Per2* locus. We further employed luciferase reporter systems and CRISPR/Cas9 genomic editing technology to demonstrate that the *Per2* upregulation by lithium is mediated through the E4BP4 transcription factor.

Previous studies have reported that clock component *Dbp* is sensitive to lithium treatments, resulting in a decreased expression [24]. Another study indicates that both *Per2* and *Egr1* (early growth response 1) are targets of lithium, while *Per2* lies downstream in the ERK/Egr1 pathway [25]. In our study, we observed downregulation of *DBP* (figure 4*m*) and upregulation of *EGR1* (electronic supplementary material, figure S8) in U2OS cells, which is consistent with previous observations that were carried out using patient-derived lymphoblastoid cells [24] and human neuroblastoma cells [25]. However, their expression in NIH3T3 cells (derived from mouse) was not affected in this study (figure 5*m*; electronic supplementary material, S8). These data indicate there may be multiple targets and multiple pathways involved in the lithium-induced effect.

Since various pathways may activate *Per2* expression directly or indirectly under the lithium treatment situation, we applied protein synthesis inhibitor CHX to U2OS cells before adding lithium. Inhibition of translation prevented the upregulation of *Per2* by lithium (figure 2*c*) and did not prevent downregulation of *E4bp4* (electronic supplementary material, figure S6). These results were consistent with our transcriptome RNA-seq results. Interestingly, three other genes being tested, *Egr1*, *Dec1* and *Dec2*, were also downregulated in the presence of CHX (electronic supplementary material, figure S7). Thus, lithium does not directly activate *Per2* promoter. Therefore, multiple cis-regulating elements that respond to different transcription factors may be downstream to the lithium treatment. Since most of the clock genes have E-box in their promoter regions and lithium was unable to induce these circadian genes' expression in the current comparative study, the E-box element was excluded from the factors that could mediate the lithium effect. *Egr1* expression was not affected in mouse NIH3T3 cells and was downregulated in human U2OS cells in the presence of CHX, indicating different pathways that lead to downstream *Per2* upregulation in human and mouse cells. Lithium not only activated *Per2* expression transiently and rapidly (figure 1*b*), but also maintained a high expression of *Per2* for relatively long time, which can be observed from the overall elevated baseline of *Per2* promoter-driven luciferase activity (figure 1*b*). In this circumstance, the period lengthening effect by lithium which was described and discussed previously [21,28] may be caused by both inhibiting GSK-3*β* and continuously activating *Per2* transcription.

As a mood-stabilizing medicine, lithium was considered to treat BPD patients partially through its effect on the circadian system [38]. It is therefore interesting that we identified both *Per2* and *E4bp4* conservatively being influenced by lithium (figures 4 and 5; electronic supplementary material, S5). As a central component in the circadian system, perturbed *Per2* expression level would affect the whole circadian clock in various aspects, including period change, amplitude and phase shift. *E4bp4* is widely expressed in peripheral tissues and regulates downstream output genes through the D-box element [10,39]. Perturbed *E4bp4* expression may further affect the amplitude of the circadian rhythm, since *E4bp4* functions as a repressive transcription factor. Recently, E4bp4 and Dec2 were reported to form a heterodimer to repress *Per2* expression through binding to the E2 element [37]. In this study, both *E4bp4* and *Dec2* were downregulated in human cells, but only *E4bp4* was downregulated in mouse cells in the presence of lithium. What is more, we found that *Dec2* was repressed both in the presence and the absence of CHX when human U2OS cells were used. We also find the E2 site rather than the B site is the preferred response element for E4bp4. These results support our findings that E4bp4 mediates the upregulation of *Per2* expression by lithium, possibly with partial help from Dec2. Thus, our findings suggest lithium can affect both the amplitude and the phase of circadian rhythms, which are key circadian clock symptoms being disrupted in the BPDs.

Both SCN and peripheral tissues show robust circadian oscillations, which involve the functions of *Per2* and *E4bp4* genes. The present study demonstrated that lithium upregulates *Per2* expression in human and murine fibroblast cells and neuronal U373 cells (figures 4 and 5; electronic supplementary material, S5). SCN belongs to the central neuron system. Therefore, lithium may affect the expressions of *Per2* in SCN. However, a solid conclusion requires further investigation. Revealing the targets of lithium will be an important step towards understanding the relationship between the circadian system and the BPD. Our translation inhibition assay with CHX indicates that *E4bp4* might be directly regulated by lithium treatments. Yet how *E4bp4* gene was downregulated in the lithium treatments requires further study. As the smallest ion, lithium may compete with other metal ions to inhibit some key enzymes in signalling pathways or key enzymes in transcription processes, such as RNA polymerase II. Furthermore, *Per2* upregulation by lithium might also be due to a smaller degradation rate of *Per2* transcript. In our future plan, we will test whether the treatment of lithium would affect the degradation rate of *Per2* with the application of a transcription inhibitor. Another important task will be to evaluate this lithium-E4bp4-Per2 axis in intact animals or in cells from BPD patients.

## Data accessibility

The data are provided in the electronic supplementary material [40].
